# Hydrogen sulfide, nitric oxide, and neurodegenerative disorders

**DOI:** 10.1186/s40035-018-0108-x

**Published:** 2018-02-13

**Authors:** Sandesh Panthi, Sumeet Manandhar, Kripa Gautam

**Affiliations:** 1 Otago School of Biomedical Sciences, University of Otago, Dunedin, New Zealand; 2 Department of Pharmacy, Chosun University, Gwangju, South Korea; 3 China Medical University, Shenyang, People’s Republic of China

**Keywords:** Hydrogen sulfide, Nitric oxide, Central nervous system, Gasotransmitters, Gaseous signaling molecules, Neurodegeneration, Neurodegenerative disorders

## Abstract

Hydrogen Sulfide (H_2_S) and Nitric Oxide (NO) have become recognized as important gaseous signaling molecules with enormous pharmacological effects, therapeutic value, and central physiological roles. NO is one of the most important regulators of the pathophysiological condition in central nervous system (CNS). It is critical in the various functioning of the brain; however, beyond certain concentration/level, it is toxic. H_2_S was regarded as toxic gas with the smell like rotten egg. But, it is now regarded as emerging neuroprotectant and neuromodulator. Recently, the use of donors and inhibitors of these signaling molecules have helped us to identify their accurate and precise biological effects. The most abundant neurotransmitter of CNS (glutamate) is the initiator of the reaction that forms NO, and H_2_S is highly expressed in brain. These molecules are shedding light on the pathogenesis of various neurological disorders. This review is mainly focused on the importance of H_2_S and NO for normal functioning of CNS.

## Background

The discovery of gaseous signaling molecules like H_2_S, NO, and Carbon monoxide (CO) added a new era in biomedical science as these molecules have great importance in mammalian physiology [1]. They have been termed as ‘gasotransmitters’ as they are either internally produced or synthesized (endogenously) in the organism or are received from the atmosphere and transmit chemical signals thereby promote or induce various physiological changes inside mammalian body [2]. The term ‘gasotransmitter’ for these molecules was firstly introduced in 2002, and these molecules share some common characteristics. They are endogenously produced, enzymatically generated, and their production can be regulated. Gasotransmitters are permeable to the cell membrane, but their functions inside the body are dependent on their concentration [3, 4]. For better understating and to boost biomedical research in the field of gasotransmitters, a society named European Network on Gasotransmitters was established in 2011.

Nitric oxide was the first gaseous molecule to be linked with its beneficial roles [5]. NO was the molecule of the year in 1992 [6] in journal ‘Science’ and was recognized by Nobel Prizes for Medicine/Physiology in 1998 [7]. Because of its toxic nature and noxious effects, beneficial roles of this molecule were previously neglected [8]. CO was the second to be discovered as neurotransmitters, and it has proved its importance in cardiovascular and neuronal functioning [9, 10]. However, the recognition of endogenous level of H_2_S in mammalian tissue, confirmed the existence of this gasotransmitters [11–13]. Synthesis, functions, and the role of these gasotransmitters in various physiological aspect is discussed in previous reviews [14–19]. The primary purpose of this review is to highlight the contextual link between CNS and these gaseous signaling molecules.

### NO and CNS

NO is synthesized in CNS from an amino acid called as L-arginine via an enzyme called NO-synthase (NOS) in equimolar amounts with L-citrulline [20, 21]. There are three different isoforms of NOS which are genetically different [22, 23]. Expression of NOS in various part of the brain is shown in Table 1. Because of its ability to passively permeate cell membrane via diffusion, there is no need of receptor binding unlike conventional neurotransmitter signaling pathways [24]. Its key potential to diffuse rapidly in aqueous and lipid environment made it unique from other CNS signaling molecules [25].

**Table 1:** Expression of NOS in various part of brain

Isoforms of NOS	Expression of NOS
eNOS	Vascular endothelium, Choroid plexus
nNOS	Neuronal cell bodies especially in thalamus, olfactory bulb, claustrum, amygdala, cortex, hippocampus, hypothalamus
iNOS	Glial cells, Macrophages, Neutrophils

NO is mainly produced by Neuronal NO-synthase (nNOS) and Endothelial NO-synthase (eNOS) under normal physiological conditions, but Inducible NO-synthase (iNOS) is only generated after induction via inflammatory mediators like cytokines and endotoxins [26]. nNOS was the first isoform to be purified and cloned from the brain [27]. NO diffuses from one neuron to another. It is not stored in any kind of synaptic vesicles, and its release is independent to membrane depolarization [25, 28]. The generation of NO is similar for all subtypes of NOS, but the functional regulation and level of production is different. nNOS and eNOS are constitutive forms of NOS, and both rely on the elevation of intracellular Ca^2+^ level to initiate NO synthesis. nNOS requires N-methyl-D-aspartate (NMDA) receptor activation, and eNOS needs calmodulin-dependent displacement of regulatory proteins for NO synthesis. However, iNOS activity is less sensitive to changes in intracellular Ca^2+^. But, it can produce a large amount of NO compared to that of NO associated with eNOS and nNOS [29].

**Fig. 1:**
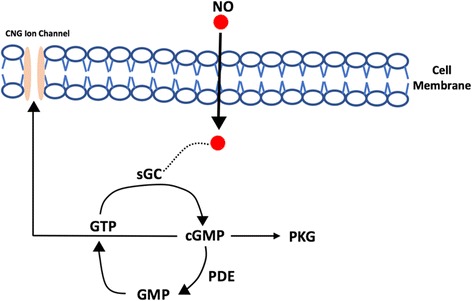
NO-cGMP signaling pathway: Highly membrane permeable NO binds with NO-soluble Guanylyl cyclase (sGC) which causes conformational changes, and it induces the conversion of GTP-cGMP. cGMP interacts with various intracellular proteins like phosphodiesterase (PDE), cGMP-gated channels (CNG) and protein kinase G (PKG) which stimulates various downstream substrates. Other various pathways are triggered, and many physiological effects starts in cellular level. (Figure adapted and modified from [31])

The signal transduction of NO in the target cell is associated with soluble Guanylate cyclase (GC)/Cyclic-guanosine mono phosphate (cGMP) (Fig. 1) or with S-nitrosylation of protein [30]. NO binds with the cGMP producing enzyme called as GC and expresses its modulating effects as pre-or post-synaptic retrograde messenger which facilitates glutamatergic neurotransmission and acts as a neuromodulator of excitatory neurotransmitter [28, 31]. Recent researches also demonstrated the effect of NO on inhibitory GABA-ergic synaptic transmission [32] via cGMP dependent suppression of potassium/chloride co-transporter [33].

Reduction of NO may lead to the inability of patients to learn and memorize due to the impairment of long-term potentiation (LTP), as NO is responsible for the increment of the synaptic efficiency of pre-synaptic glutamatergic neurons and this increment induces LTP [34]. This gaseous signaling molecule also exerts protective role in brain-ischemia reperfusion injury as a result of its strong stimulatory effect on angiogenesis and vasodilation [35].

Hemodynamic and vasodilation activity of NO donors [*S*-nitrosoglutathione (GSNO), *S*-nitroso-*N*-acetyl-penicillamine (SNAP), sodium nitroprusside (SNP), methylamine hexamethylene methylamine NONOate (MAHMA), propylamine propylamine NONOate (PAPA), 3-morpholinosydnonimine (SIN-1), and nitroglycerin (NTG)] has also provided cerebrovascular neuroprotective role in the various experimental model of stroke [36, 37]. NO produced from endothelial cells and adrenergic neurons regulates cerebral blood flow and smooth muscle tone during conditions like hypoxia, hypercapnia, hyperoxia, etc. eNOS mediates the basal release of NO to regulate the cerebral blood flow in various mammals. This role of NO has been confirmed by various recent researches using NOS inhibitors [38–40].

Age-related decrease in cGMP was also linked with increased NOS level in one recent study which may be helpful for anti-aging therapies [41]. NO is also suspected to be a crucial molecule to nitrate/S-nitrosylate brain-derived neurotrophic factor (BDNF) and tropomyosin-related kinase receptor which helps the maintenance of synaptic plasticity and LTP [42]. A study has found that NO directly activates ryanodine receptor (RyR), which cause the intracellular release of Ca^2+^ to towards CNS, and it is believed to promote the prolonged Ca^2+^ signaling in the brain. Alike, BDNF and tropomyosin-related kinase receptor, this process is also triggered by reversible *S*-nitrosylation that cause the Ca^2+^ release. This whole process is essential for cerebellar synaptic plasticity [43]. Role of NO in maintaining cerebellar synaptic plasticity, synaptic transmission efficiency, and cerebellar LTP are also studied and mentioned [44–46].

NO was also found to have affect in the sleep-wake cycle. Intraperitoneal (i.p.) administration of nNOS inhibitor caused the drop in rapid eye movement (REM) and sleep-wave-sleep. This relationship between the production of NO and sleep is also thought to be linked with various neurodegenerative disorders [47]. Researchers linked the role of NO with experimental seizure model where overstimulation of NMDA receptors is believed to cause the prolonged release of NO [48]. However, the overactivity of same NMDA receptors leading overproduction of NO may contribute to cell death-initiating various neurodegenerative conditions like Alzheimer’s disease (AD), Parkinson’s disease (PD), amyotrophic lateral sclerosis (ALS), stroke and inhibition of NOS could be neuroprotective [49].

Brain hypoperfusion and increased vascular oxidative stress are the common phenomena involved in AD [50, 51]. Few researchers also found that abnormal nNOS expressions are early symptoms of the AD and cognitive impairment involved in AD [52, 53]. It was found that imbalance between the nitrotyrosine and all three isoforms of NOS results in the increased amount of nitrosylation and oxidative products in blood and CSF of AD patients [54]. Some more physiological roles of the different isoform of NOS are listed in Table 2.

**Table 2:** Physiological roles of different isoform of NOS

Isoforms of NOS	Functions
eNOS	• Preservation and maintenance of brain’s microcirculation [21].
	• Inhibition of platelet aggregation [131].
	• Reduction of smooth muscle proliferation [21, 39]
nNOS	Important roles in memory formation, CNS blood flow, neuronal plasticity, transmission of pain signals [21].
iNOS	Response to proinflammatory cytokines or endotoxins [21].

Stroke (ischemic stroke) is also characterized by interruption of blood flow in the cerebral artery which ultimately result in ischemia and tissue death. Using the experimental animal model of stroke, Huang et al. in 1996 found that eNOS deficient mice has bigger infracts than wild type [55]. eNOS was also found to maintain the level of cerebral blood flow (CBF) after traumatic brain injury (TBI). However, eNOS knock-out mice has greater reduction of CBF after TBI [56, 57]. Various studies have also proved that NO depletion has a critical role in cortical spreading depression, early brain injury, microthrombus formation, changes in blood flow after subarachnoid hemorrhage and if we can target this pathway, then it is possible to prevent secondary neuronal injury [21].

Interestingly, inhaled and intravenous (i.v.) injection of sodium nitrite (NO donor) was found to have neuroprotective role in mice and rats during cardiac arrest (seen in the clinical model of ischemia/reperfusion) [58, 59]. Not only in the mice model of ischemia, but there are also few studies conducted till now which support that inhaled NO or i/v injected of L-arginine or sodium nitrite has protective outcomes in the development of brain after injury, using various experimental injury models [60–67]. This gasotransmitter is also associated to normalize capillary blood flow, improvement of delivery of oxygen [68], prevention and reversal of cerebral vasospasm [21, 66, 67], mitochondrial respiration [58], development of healthy brain [21], myelination [69], and protective role after peripheral nerve injury [15].

However, evidence suggests that higher concentration of NO can impact neuronal death in several ways. Neuronal death may occur due to energy depletion-induced necrosis that may stop mitochondrial respiration or slow inhibition of glycolysis. So, the role of NO is particularly dependent on its concentration, time-course exposure, and presence/absence of ROS at particular level and cells. Thus, being a neuroprotectant at low level, NO might behave as toxicant at higher concentration [70]. It was found that after the administration of nitro-L- arginine, the infarct size of nNOS-KO mice became larger (whereas vascular NO protects after middle cerebral artery occlusion) and NO induced calcium release was found to be involved in neuronal cell death [43, 71]. Because of this reason NO is often termed as a “double-edged sword” [48]. One study revealed the critical role of NO during neurodegenerative disorders and brain aging can form blood-cerebrospinal fluid barrier and this may interfere choroid-plexus gateway activity [72]. Similarly, another finding stated NO as the negative player in the progression of pathological nature of AD [73]. Recently, another deleterious role of NOS with involvement of CNS in mouse model of dengue has been discovered [74]. NO is involved in various physiological functioning which is explained earlier in this review, however, if the physiological control of signaling pathways involved with NO or NOS fails, then NO and other reactive nitrogen species (RNS) can cause neuroinflammation and neurodegeneration [75].

### H_2_S and CNS

H_2_S is a toxic and poisonous gas having an odour of rotten eggs. Alike other gasotransmitters, the physiological role of H_2_S was overlooked or not paid attention due to its toxicity [76]. It is the most recent gaseous signaling molecule discovered after NO and CO having enormous pathophysiological significance in various disease and conditions [77]. It is sulfur analog of water, and because of its weak intermolecular force, it exists in gaseous form [78]. It is synthesized via both enzymatic and non-enzymatic pathways inside mammalian tissue, but non-enzymatic route accounts for a small portion. Cystathionine β-synthase (CBS) and Cystathionine γ-lyase (CSE) are two enzymes responsible for biosynthesis of H_2_S from L-cysteine [79, 80]. 3-mercaptopyruvate sulfurtransferase (3MST) is another enzyme that can generate H_2_S through cys-catabolism pathway. CSE and CBS are localized in the cytoplasm of cell, but 3MST is expressed partly in mitochondria and cytoplasm [81, 82]. A recent study showed H_2_S could be produced from D-cysteine via enzyme D-amino acid oxidase (DAO) [83, 84]. Non-enzymatically, it can be produced from thiosulfate [14] and glucose (via glycolysis) or from phosphogluconate via NADPH oxidase [85, 86]. Although H_2_S has beneficial roles in various hematologic diseases, urological disease, cardiovascular functioning and oxidative stress, the effect of H_2_S in CNS has attracted a lot of attention over the past few years [14, 77, 87]. Expression of different H_2_S producing enzymes in various parts of mammalian tissues is listed in Table 3 [88]. Important signaling events of H_2_S in various neuronal cells/cell lines are listed below [89]:

1. Inhibition of monoamine oxidase (via catecholamines)
2. NMDA potentiation (via glutamate)
3. Cystic fibrosis transmembrane conductance regulator (CTFR) channel activation (via chloride channels)
4. K_ATP_ and K_Ca2+_ channel activation (via potassium channels)
5. Intracellular calcium mobilisation, L-type and T-type channel activation (via calcium channels)
6. Supression of various types of neuronal toxicity (via oxidative stress)
7. Inhibition of p38-MAPK (via mitogen and tyrosine kinase receptors)
8. Stimulation of PKA and elevation of cAMP (via PKA)

**Table 3:** Expression of different H_2_S producing enzymes in various parts of mammalian tissues

H_2_S producing enzymes	Expression
CSE	Liver, Kidney, Aorta, Ileum but weakly found in brain.
CBS	Liver, Kidney, and Brain (astrocytes)
3MST	Liver, Kidney, Heart, Brain (Purkinje cells of cerebellum, pyramidal neurons of cerebellar cortex, hippocampus, mitral cells of olfactory bulb, retinal neurons), Vascular endothelium, Smooth muscle.

AD, a common form of dementia, characterized by memory impairment, personality changes, and various neuropsychiatric symptoms which cause neuronal apoptosis, neuronal inflammation (induced by amyloid-β), and increased oxidative stress [90–93]. Level of H_2_S in the brain of patient with AD is lower than healthy people of same age [94]. A recent study revealed that in a rat model of vascular dementia, plasma H_2_S level was lower and i.p. injection of NaHS (H_2_S donor) protected neuronal injury and improved behavioral (learning and memory) tests results [95]. Another study demonstrated that progression of AD was abrupted after treatment with spa-water rich of H_2_S content [96]. Role of H_2_S in the improvement of cognitive functioning, spatial learning and memory [96], and neuroprotective effects [14, 90, 93, 97] is also providing us hopes against the AD.

PD is characterized by progressive degeneration of dopaminergic neurons in the substantia nigra of midbrain which is age-related and leads to the formation of Lewy bodies in soma of residual neurons [77, 98]. Previous studies based on animal models found that inhalation or injection of H_2_S donors prevented abnormalities related to PD (microglial activation or motor dysfunction) including neuroprotective, neuromodulatory, and therapeutic roles of H_2_S in PD [99–101]. H_2_S-mediated anti-oxidative, anti-inflammatory, anti-apoptotic, and pro-survival effects linked with PD is also reviewed in recent paper [102].

TBI is one of the most common causes of death among youth in today’s world and is considered as a public health epidemic. Memory impairment and cognitive dysfunction are two immediate effects of TBI whereas rapid and extreme production of ROS are also associated with secondary neuronal injury after TBI [103–106]. Karimi et al. injected NaHS intraperitoneally and observed neuroprotective effect of H_2_S in TBI induced impaired memory in rats [103]. Zhang et al. found H_2_S as a neuromodulator by injecting same H_2_S donor which decreased TBI induced lesion volume in brain [107]. NaHS proved to be the neuroprotective in various other pathological conditions [108–112]. These studies are also supported by recent finding which showed dynamic changes in CBS and H_2_S levels in various part of the brain in experimental TBI models [107].

Huntington’s Disease (HD) is associated with neurotoxicity, behavioural changes, impairment of motor coordination, and oxidative stress. Paul et al. showed that there is a reduction of level of CSE in mammalian tissues with HD. They demonstrated that loss of CSE mediates degeneration of neuronal cells and progression of HD [113, 114]. Studies have shown that patients with Down Syndrome (DS) has the higher level of CBS compared to that of a normal individual. This overexpression of CBS is believed to be the cause of abnormal cognitive ability in children with DS and may lead to AD in DS adults. Overproduction of H_2_S is also associated with ethylmalonic encephalopathy [115]. Some other neuroprotective [40, 78, 116–118], neurotransmissive role (facilitation of the induction of hippocampal LTP) [119, 120] of H_2_S and its role in protection of neurons from apoptosis, degeneration [121, 122] and oxidative stress [121] are also studied extensively and illustrated in Table 4.

**Table 4:** Physiological functions of H_2_S based on its neuroprotective and neuromodulatory effects

Mode of physiological functions of H_2_S	Evidences
Neuroprotection	PD: Inhibits oxygen consumption and 6-hydroxydopamine evoked NADPH oxidation.
	Acts on various protein kinases.
	HD: Upregulation of GSH enzyme and reveals the learning and memory problem.
	AD: Decreases protein oxidation and lipid peroxidation.
	Reduces homocysteine-induced toxicity.
	Influences synaptic remodelling.
	ALS: Proper regulation of GSH enzyme and reduction of oxidative stress.
	TBI: Protection via apoptotic and autophagic pathway.
	Protective effects against neuropathic pain and brain edema.
Neuromodulation	• Long term potentiation.
	• CFTR Cl^−^ and K_ATP_ cycle regulation.
	• Enhancement of NMDA receptor activity.
	• Regulation of intracellular Ca^2+^.

### Interrelationship between NO and H_2_S

Various studies have shown that these gasotransmitters potentiate or antagonize each other’s effect in production, downstream of certain molecular target, and direct chemical interaction [123]. These gasotransmitters share the same signaling pathway in the regulation of angiogenesis and endothelium dependent vasorelaxation [124]. Few articles demonstrated the common pathway of these gasotransmitters in the mammalian cardiovascular system [2, 17, 125, 126]. Additionally, NO and CO are also found to have link in vasorelaxation and stimulation of calcium sensitive potassium channels [123, 127].

Gasotransmitters also have a tendency to compete with each other. CO and NO have particular relationships with CBS. Research suggests that NO can block the enzymatic activity of H2S via binding with CBS and CBS has also high preference for CO. If eNOS is impeded, then it cancels out the angiogenic effects of H_2_S whereas blocking H_2_S significantly lowers the angiogenic effects of NO. CO and H_2_S act on the same molecular target but have opposite results. Even though H_2_S, NO, and CO compete and share similar signaling pathways with each other, their interactions provide beneficial effects on mammalian physiology [14, 124, 128–130].

## Conclusions

Current understandings and the published data in this field has made clear about their importance in the mammalian physiology and pathology. There are still many controversies surrounding the signaling pathways, beneficial roles, and harmful effects of these gasotransmitters. However, their role in mediation and modulation of cell-to-cell communication is globally accepted fact. Research and studies regarding these molecule is still in the preliminary stage in the field of biomedical science, and especially their role in CNS is relatively unexplored. Further research also should be focused on their combinatorial effect and signaling pathways regarding their antagonistic effect should be disclosed for the development of new therapeutic approaches for various neurological disorders.

## Abbreviations

CBS: Cystathionine β-synthase
CSE: Cystathionine γ-lyase
3MST: 3-Mercapto pyruvate sulfurtransferase
AD: Alzheimer’s disease
ALS: Amyotrophic lateral sclerosis
BDNF: Brain derived neurotrophic factor
cAMP: Cyclic adenosine mono phosphate
CBF: Cerebral blood flow
CNS: Central nervous system
CO: Carbon mono-oxide
CTFR: Cystic fibrosis transmembrane conductance regulator
DAO: D-amino acid oxidase
DS: Downs syndrome
eNOS: Endothelial nitric oxide synthase
GC: Guanylyl cyclase
H_2_S: Hydrogen sulfide
HD: Huntington’s disease
i.p.: Intraperitoneal
i.v.: Intravenous
iNOS: Inducible nitric oxide synthase
LTP: Long term potentiation
MAPK: Mitogen-activated protein kinase
NADPH: Nicotinamide adenosine dinucleotide phosphate
NMDA: N-methyl-D-aspartate
nNOS: Neuronal nitric oxide synthase
NO: Nitric oxide
NOS: Nitric oxide synthase
PD: Parkinson’s disease
PKA: Protein kinase A
REM: Rapid eye movement
RyR: Ryanodine receptor
TBI: Traumatic brain injury
